# Machine learning prediction of adverse events during minutes 15–30 of recovery in patients event-free at 15 min after sedated gastrointestinal endoscopy

**DOI:** 10.3389/fmed.2026.1930976

**Published:** 2026-08-27

**Authors:** Siyuan Huang, Jiayao Zhang, Lu Yin, Meiyuan Luo, Anna Jia, Dihong Chen

**Affiliations:** 1 Department of Anesthesiology, West China Hospital, Sichuan University, Chengdu, China; 2 West China School of Nursing, Sichuan University, Chengdu, China

**Keywords:** adverse events, machine learning, predictive model, sedated gastrointestinal endoscopy, shapley additive explanations, XGBoost

## Abstract

**Background:**

Sedated gastrointestinal (GI) endoscopy is widely used for diagnosis and treatment but may be complicated by respiratory and hemodynamic adverse events (AEs) during post-anaesthesia recovery. Early risk identification may support targeted monitoring and efficient allocation of recovery-care resources. This study aimed to develop and internally evaluate machine-learning (ML) models for identifying respiratory and hemodynamic AEs occurring during 15–30 min after awakening following sedated GI endoscopy.

**Methods:**

Consecutive patients undergoing sedated GI endoscopy at West China Hospital, Sichuan University, between September 2023 and April 2024 were enrolled. Predictors were selected using the Boruta algorithm. Six supervised ML models—decision tree (DT), random forest (RF), multilayer perceptron (MLP), support-vector machine (SVM), extreme gradient boosting (XGBoost), and light gradient boosting machine (LightGBM)—were trained and evaluated. Performance was assessed using the area under the receiver operating characteristic curve (ROC-AUC), area under the precision-recall curve (PR-AUC), Brier score, calibration intercept and slope, calibration plots, classification metrics, and decision-curve analysis (DCA). SHapley Additive exPlanations (SHAP) were used to interpret the final model.

**Results:**

Among 1,070 included patients, 103 (9.6%) experienced the prespecified AE outcome. XGBoost achieved the highest cross-validated training ROC-AUC of 0.971 (95% CI, 0.956–0.986) and a ROC-AUC of 0.910 (95% CI, 0.834–0.987) in the independent validation set. The validation PR-AUC and Brier score were 0.815 and 0.076, respectively; the calibration intercept was −1.760 and the slope was 1.480. The final model included heart-rate change, SpO_2_ change, baseline mean arterial pressure, sufentanil dose, and intra-procedural metaraminol use. SHAP analysis quantified each predictor's contribution to model output but was not interpreted as evidence of causality or clinical modifiability.

**Conclusions:**

XGBoost showed promising discrimination for identifying respiratory and hemodynamic AEs during early recovery. However, prospective studies with temporally separated predictor and outcome windows and external validation are required before clinical implementation.

## 1 Introduction

Emerging non-invasive and minimally invasive approaches are increasingly being investigated for gastric cancer detection and risk stratification. Metabolomics-based ML models, including explainable frameworks based on plasma metabolomic profiles, have shown potential for non-invasive gastric cancer detection. Tongue-coating metaproteomics represents a minimally burdensome approach for identifying individuals at increased risk, while optimized ML algorithms may further improve biomarker-based gastric cancer classification (1–4). These approaches may ultimately support population screening, risk stratification, or pre-endoscopic triage by identifying individuals who are more likely to benefit from further endoscopic evaluation.

These emerging approaches address a different stage of care from GI endoscopy and should be considered complementary rather than competing strategies. When clinically indicated, GI endoscopy enables direct mucosal visualization, targeted biopsy, histopathological confirmation, and endoscopic intervention (5). A potential integrated pathway may therefore involve non-invasive or minimally invasive screening, risk stratification, referral for endoscopy, endoscopic diagnosis or treatment, and peri-procedural safety management. The present study focuses on the final stage of this pathway and is not intended to detect gastric cancer or determine whether a patient should undergo endoscopy.

The increasing use of GI endoscopy, together with population ageing and the expansion of screening programmes, has placed growing pressure on procedural safety and healthcare-resource allocation. A survey estimated that the annual number of GI endoscopic procedures in China could approach 51 million by 2030 (6). Sedation is increasingly used to improve patient comfort, procedural tolerance, cooperation, and examination completion (7, 8). However, sedative and analgesic medications may also contribute to cardiopulmonary AEs, including hypoxaemia, respiratory depression, airway obstruction, hypotension, and cardiac arrhythmias (9, 10). The reported incidence of these events varies substantially according to patient characteristics, procedure type, sedation depth, monitoring strategy, and outcome definition (10, 11). Although many events are transient, they may require airway support, supplemental oxygen, vasoactive medication, prolonged monitoring, or escalation of care (10, 11).

The post-anaesthesia care unit (PACU) represents an important transition between residual sedative effects and the recovery of consciousness, respiratory drive, airway patency, and cardiovascular stability. Respiratory and haemodynamic AEs may newly emerge or persist during this early recovery period because of residual medication effects, incomplete restoration of ventilation, upper-airway obstruction, underlying comorbidities, and procedure- or treatment-related physiological disturbances (10–12). Early identification of patients at increased risk may therefore support risk-adapted monitoring and more efficient allocation of PACU resources.

With the increasing availability of peri-procedural clinical and physiological data, ML methods offer a potential approach for modelling nonlinear relationships and complex interactions among multiple predictors (13, 14). Conventional regression methods remain clinically valuable, but ML algorithms may provide additional flexibility when analysing complex data structures (13). Nevertheless, the limited interpretability of some models and uncertainty regarding their integration into clinical workflows may hinder clinical acceptance and implementation (13, 15). Interpretable approaches such as SHapley Additive exPlanations can quantify the contribution of individual predictors to model-estimated risk and provide both global and patient-level explanations of tree-based models (14, 16).

Accordingly, this prospective observational study aimed to develop and internally evaluate multiple ML models for predicting newly occurring respiratory or haemodynamic AEs during PACU recovery after sedated GI. Using 15 min after awakening as the reference point for outcome definition, the models were developed to identify patients who experienced newly occurring AEs during 15–30 min after awakening. SHAP analysis was used to describe the contribution of individual predictors to the final model predictions.

## 2 Materials and methods

### 2.1 Inclusion and exclusion criteria

A prospective study was conducted at the Digestive Endoscopy Center of West China Hospital, Sichuan University, from September 2023 to April 2024, adhering to the Transparent Reporting of a Multivariable Prediction Model for Individual Prognosis or Diagnosis plus Artificial Intelligence (TRIPOD + AI) statement (17). Eligible participants included individuals of any sex aged 18–60 years, American Society of Anesthesiologists(ASA) anesthesia risk classification I-III, who were receiving propofol combined with sufentanil intravenous anesthesia during surgery, who possessed intact cognitive function, and who provided informed consent. The exclusion criteria included patients with severe preexisting cardiopulmonary, hepatic, or renal dysfunction at data collection; symptoms of upper respiratory tract infection; or any cause resulting in a PACU stay <30 min. The study was approved by the Ethics Committee of West China Hospital of Sichuan University [Grant Number:2023(1619)] and registered with the China Clinical Trial Registry (Registration Number: ChiCTR2300077983). Before participation in the study, all patients provided written informed consent. The study followed the ethical principles outlined in the Declaration of Helsinki.

### 2.2 Sample size

A *post hoc* sample-size adequacy assessment was performed using the pmsampsize package in R for a binary-outcome prediction model (18). Assuming an adverse-event proportion of 9.5%, five candidate predictor parameters, an anticipated Cox-Snell R^2^ of 0.23, and a target global shrinkage factor of 0.90, the estimated minimum development sample size was 186 patients, corresponding to approximately 18 outcome events. The required sample sizes under the three criteria were 170, 186, and 133 patients, respectively, and the largest estimate was adopted.

### 2.3 Patient monitoring and sedative intervention

All patients underwent routine preoperative fasting and GI preparation, including fasting for 8 h and abstaining from fluids for 4 h. Intravenous access was established using a 22-gauge catheter approximately 30 min before the procedure. In the examination room, patients were positioned in the left lateral decubitus position, fitted with a bite block, and monitored continuously for heart rate (HR), systolic blood pressure (SBP), diastolic blood pressure (DBP), and oxygen saturation (SpO_2_). Oxygen was administered via nasal cannula at 3 L/min. Sedated GI was performed under intravenous sufentanil and propofol. Sufentanil was generally administered at a target dose of 0.1 µg/kg according to body weight, and propofol was administered at 1.0–1.5 mg/kg. The actual sufentanil could be adjusted according to the patient's clinical condition and anesthesiologist judgment and was rounded to a clinically feasible dose. Sufentanil was supplied in 10 µg ampoules, and the calculated dose was rounded to a clinically feasible dose before administration. For modelling purposes, the sufentanil variable represented the actual absolute induction dose administered, expressed in µg. Endoscopy was initiated after loss of the eyelash reflex and adequate sedation. Additional propofol (0.2–0.5 mg/kg) was administered when clinically indicated by patient movement or inadequate sedation. After completion of the procedure, patients were transferred to the PACU under the supervision of the gastroenterologist and anesthesiologist. Capillary blood glucose was measured 30 min after entry into the procedure room using a point-of-care glucose meter in accordance with the institutional standard operating procedure. Recovery was assessed every 15 min using the modified Aldrete score, and a score of ≥9 was used as the criterion for PACU discharge.

### 2.4 Data collection and definitions

On the basis of a review of relevant literature and clinical observations from previous studies, a set of factors potentially influencing AEs during sedated digestive endoscopy was identified. We designed a questionnaire to collect relevant data. Trained investigators recruited patients. Data were collected according to their measurement time relative to the prespecified 15-minute reference point after awakening. Pre-procedural variables obtained before sedation included age, sex, body mass index (BMI), ASA class, alcohol consumption, smoking, vertigo, hypertension, diabetes mellitus (DM), heart disease, lung disease, electrocardiogram, baseline mean arterial pressure (MAP), and baseline clinical conditions or symptoms, including reflux misabsorption, nausea, emesis, dysphoria, ventosity, hypoglycaemia, pain, and giddy. Baseline MAP was defined as the first stable MAP recorded before the administration of sedative or anaesthetic medication. Intra-procedural variables included the total doses of propofol and sufentanil, atropine, metaraminol, endoscopic operation time, and GI endoscopy. Early PACU variables included awakening time, HR change, SpO_2_ change, and Breathe change. Awakening time was defined as the interval between the completion of the endoscopic procedure and the patient's first appropriate response to verbal commands. HR change, SpO_2_ change, and breathe change were calculated as the relative changes between measurements obtained at 15 and 30 min after awakening, using the 15-minute measurements as the reference values (Supplementary Table S1).

AEs were defined as a composite of new-onset respiratory depression and haemodynamic events occurring between 15 and 30 min after awakening. The 15-minute time point was prespecified as the reference point for outcome assessment. Accordingly, the outcome analysis focused on newly occurring AEs during 15–30 min among patients who were event-free at 15 min; events occurring within the first 15 min after awakening were not included in the predefined outcome. Respiratory depression was defined as a respiratory rate <8 breaths/min and/or SpO_2_ < 90% (19). Once respiratory depression occurs, mask-assisted ventilation with positive pressure should be immediately initiated, and endotracheal intubation should be performed if necessary. A hemodynamic event was defined as an intraoperative decrease in MAP and/or HR exceeding 20% of baseline values or a SBP ≤80 mmHg. Upon the occurrence of a hemodynamic event, fluid therapy should be initiated immediately. If fluid resuscitation was inadequate, vasoactive agents were administered (Supplementary Table S2).

### 2.5 Data preprocessing

The complete dataset was first divided into a training set and an independent validation set at a ratio of 7:3 using stratified random sampling according to AE status. A fixed random seed of 915 was used. After data splitting, the independent validation set was locked and was not used during data preprocessing, feature selection, class balancing, cross-validation, hyperparameter tuning, model selection, or classification-threshold determination (17, 20, 21).

All preprocessing and model-development procedures were performed using the training set. Missing values occurred only in continuous variables, with less than 10% missingness for each affected variable; no missing values were observed in categorical predictors. K-nearest-neighbor (KNN) imputation was fitted once using the complete training set. Five nearest neighbors and Gower distance were used (20, 22, 23). All available predictor variables were considered when identifying neighboring observations, whereas AE status was excluded from the imputation procedure (22, 24). No separate centering or scaling was performed before imputation. The training-derived imputation procedure was subsequently applied to the validation set without refitting.

Model development and hyperparameter tuning were conducted within the imputed training set using non-repeated stratified five-fold cross-validation. Because the training and held-out validation sets were derived from the same clinical cohort, this procedure was regarded as internal rather than external validation.

### 2.6 Feature selection and class-imbalance handling

Following KNN imputation, Boruta feature selection was performed once using the complete imputed training set before cross-validation. Boruta was implemented with maxRuns = 100, a significance level of 0.01, Bonferroni adjustment for multiple comparisons, and a random seed of 42. Variables remaining Tentative were resolved using TentativeRoughFix(), and predictors ultimately classified as Confirmed were retained. The resulting predictor set was fixed and used throughout subsequent cross-validation and model development. The validation set was not used during feature selection.

Class imbalance was subsequently addressed using Synthetic Minority Over-sampling Technique for Nominal and Continuous features (SMOTENC), which accommodates mixed continuous and categorical predictors (25). During each iteration of stratified five-fold cross-validation, SMOTENC was applied exclusively to the analysis fold using five nearest neighbors, Gower distance, an oversampling ratio of 1, and a fixed random seed of 42. The corresponding assessment fold was not oversampled and retained its original outcome distribution.

Models were fitted and hyperparameters were tuned using the resampled analysis folds, whereas performance was evaluated using the original, non-oversampled assessment folds. The validation set retained its original outcome distribution and was not involved in imputation fitting, feature selection, oversampling, hyperparameter tuning, model selection, or threshold determination.

### 2.7 Model development and comparison

Six ML algorithms were evaluated: DT (26), RF (27), MLP (28), SVM (29), XGBoost (30), and LightGBM (31). Hyperparameters were optimized using random grid search within the five-fold cross-validation procedure. The search ranges and final parameter values were reported (Supplementary Table S3). Candidate models were compared using the mean cross-validated ROC-AUC as the prespecified primary model-selection criterion. Model selection was conducted exclusively within the training set. XGBoost was selected as the final model because it achieved the highest mean ROC-AUC during cross-validation; performance in the independent test set was not considered during model selection, hyperparameter tuning, or classification-threshold determination.

Model performance was evaluated in both the training and independent test sets. Discrimination was assessed using the ROC-AUC and PR-AUC. Threshold-dependent classification performance was evaluated using accuracy, balanced accuracy, F1 score, J-index, kappa coefficient, Matthew correlation coefficient (MCC), positive predictive value (PPV), negative predictive value (NPV), Precision, Recall, sensitivity (SENS), and specificity (SPEC). The optimal classification threshold was determined from the out-of-fold predicted probabilities in the training set by maximizing the Youden index and was subsequently fixed before evaluation of the independent test set.

Model calibration was assessed using calibration plots, the Brier score, calibration intercept, and calibration slope. A calibration intercept close to 0 and a calibration slope close to 1 indicated satisfactory agreement between the predicted and observed risks. The Brier score quantified the overall accuracy of the predicted probabilities, with lower values indicating better probabilistic performance. Finally, DCA was performed across a clinically relevant range of threshold probabilities to evaluate the net clinical benefit of each model compared with the default strategies of treating all patients or treating no patients.

A conventional multivariable logistic regression model was developed as a reference using the same five predictors, data partition, and training-derived KNN imputation procedure as the final XGBoost model. The model was fitted to the original imputed training set without SMOTENC or hyperparameter tuning. XGBoost and logistic regression were evaluated using the prespecified discrimination, classification, calibration, and DCA. Model-specific thresholds were determined in the training set using the Youden index and fixed before independent validation.

### 2.8 Model interpretability

SHAP values were calculated for the final XGBoost model. For each patient, SHAP values decomposed the difference between the model output and its expected value into additive contributions from the individual predictors. Global feature importance was quantified as the mean absolute SHAP value across all patients, with larger values indicating a greater overall contribution to the model predictions (32). Positive and negative SHAP values indicated contributions toward higher and lower model-predicted risks of subsequent AEs, respectively. SHAP values were interpreted as model-based feature contributions reflecting the associations learned by the prediction model and not as evidence of causal effects, protective effects, or treatment effects.

### 2.9 Statistical analysis

Data analysis was performed via R version 4.4.2. Continuous variables were assessed for normality using the Kolmogorov–Smirnov (K–S) test and visual inspection of their distributions. Non-normally distributed variables were summarized as median and interquartile range [M(Q1, Q3)] and compared using the Mann–Whitney U test. Categorical variables were presented as numbers and percentages and compared using the chi-square test or Fisher's exact test, as appropriate. All tests were two-sided, and a *P* value <0.05 was considered statistically significant.

Where the number of events permitted, exploratory sensitivity analyses were performed for respiratory and hemodynamic AEs separately. These analyses used the same outcome window and the same training-validation allocation as the primary analysis. Because the subtype-specific event counts were limited, the results were interpreted as exploratory and were not used to modify the final model or its hyperparameters.

## 3 Results

### 3.1 Patient characteristics

**Figure 1:**
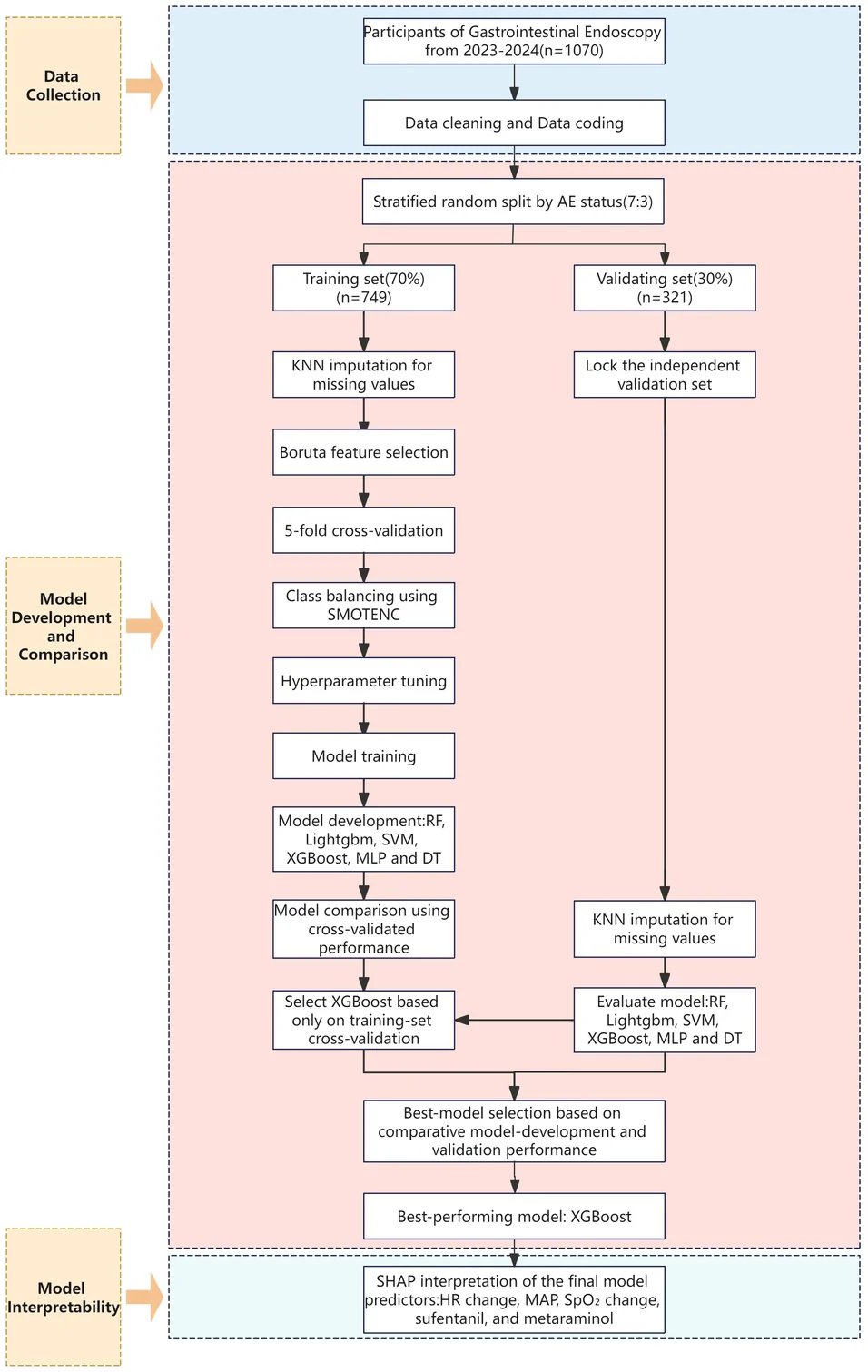
Study flow of predictors and subsequent PACU AEs.

The entire research design process is shown in Figure 1.

### 3.2 Basic characteristics of the training and validation sets

Among the 1218 initially eligible patients, 148 experienced an AE within the first 15 min after awakening and were excluded. Among the 1,070 patients who were event-free at 15 min after awakening, 103 (9.6%) experienced a newly occurring AE during 15–30 min after awakening. The dataset was divided into a 70% training set (*n* = 749 patients) and a 30% validation set (*n* = 321 patients). Within the training set, AEs occurred in 9.7% (73 patients) of patients who underwent sedated endoscopy, whereas this proportion was 9.3% (30 patients) in the validation set. The basic characteristics categorized by training and validation sets are summarized in Table 1. The mean actual sufentanil dose was 4.536 µg in the training set, 4.464 µg in the validation set, and 4.514 µg overall, with corresponding ranges of 0–10, 0–8, and 0–10 µg. Although both the median and interquartile range were 5.00 µg in the original descriptive analysis, a small proportion of patients received different doses because the weight-based target dose of 0.1 µg/kg was rounded to a clinically feasible dose. Mean pain scores were 0.23, 0.21, and 0.23, with corresponding ranges of 0–4, 0–3, and 0–4, respectively.

**Table 1:** Demographic and clinicopathologic variables within the entire cohort stratified into training and validation sets.

Variable	Total (*n* = 1,070)	Training (*n* = 749)	validation(*n* = 321)	*P* value
Age, Median (Q1, Q3)	52.00 (40.00–60.00)	52.00 (40.00–60.00)	52.00 (42.00–60.00)	0.609
BMI, Median (Q1, Q3)	22.67 (20.76–24.97)	22.66 (20.76–24.97)	22.64 (20.85–24.99)	0.859
Propofol, Median (Q1, Q3)	67.50 (50.00–90.00)	60.00 (50.00–90.00)	70.00 (50.00–90.00)	0.555
Sufentanil, Median (Q1, Q3)	5.00 (5.00–5.00)	5.00 (5.00–5.00)	5.00 (5.00–5.00)	0.701
Endoscopic operation time (Q1, Q3)	20.00 (15.00–25.00)	20.00 (15.00–25.00)	20.00 (15.00–25.00)	0.071
MAP, Median (Q1, Q3)	95.00 (88.67–101.00)	95.00 (88.67–101.00)	95.00 (88.33–100.67)	0.865
Awakening time, Median (Q1, Q3)	20.00 (15.00–25.00)	20.00 (15.00–25.00)	20.00 (15.00–26.00)	0.646
Pain, Median (Q1, Q3)	0.00 (0.00–0.00)	0.00 (0.00–0.00)	0.00 (0.00–0.00)	0.851
HR change, Median (Q1, Q3)	0.07 (0.03–0.11)	0.07 (0.03–0.11)	0.07 (0.03–0.12)	0.662
SpO2 change, Median (Q1, Q3)	0.01 (0.00–0.02)	0.01 (0.00–0.02)	0.10 (0.00–0.02)	0.178
Breathe change, Median (Q1, Q3)	0.09 (0.06–0.14)	0.90 (0.06–0.14)	0.90 (0.06–0.15)	0.284
Gender, *n* (%)				0.861
Male	471 (44.02)	331 (44.19)	140 (43.61)	
Female	599 (55.98)	418 (55.81)	181 (56.39)	
Smoking, *n* (%)				0.987
Never smoker	833 (77.85)	583 (77.84)	250 (77.88)	
Current smoker	237 (22.15)	166 (22.16)	71 (22.12)	
Alcohol consumption, *n* (%)				0.740
Never drinker	741 (69.25)	521 (69.56)	220 (68.54)	
Current drinker	329 (30.75)	228 (30.44)	101 (31.46)	
ASA class, *n* (%)				0.494
I	88 (8.22)	63 (8.41)	25 (7.79)	
II	931 (87.01)	654 (87.32)	277 (86.29)	
III	51 (4.77)	32 (4.27)	19 (5.92)	
Vertigo, *n* (%)				0.802
No	1,042 (97.38)	730 (97.46)	312 (97.20)	
Yes	28 (2.62)	19 (2.54)	9 (2.80)	
DM, *n* (%)				0.625
No	1,009 (94.30)	708 (94.53)	301 (93.77)	
Yes	61 (5.70)	41 (5.47)	20 (6.23)	
Heart disease, *n* (%)				0.390
No	1,051 (98.22)	734 (98.00)	317 (98.75)	
Yes	19 (1.78)	15 (2.00)	4 (1.25)	
Hypertension, *n* (%)				0.359
No	970 (90.65)	683 (91.19)	287 (89.41)	
Yes	100 (9.35)	66 (8.81)	34 (10.59)	
Lung disease, *n* (%)				1.000
No	940 (87.85)	658 (87.85)	282 (87.85)	
Yes	130 (12.15)	91 (12.15)	39 (12.15)	
Electrocardiogram, *n* (%)				0.940
Normal	989 (92.43)	692 (92.39)	297 (92.52)	
Abnormal	81 (7.57)	57 (7.61)	24 (7.48)	
Atropine, *n* (%)				1.000
No	1,064 (99.44)	745 (99.47)	319 (99.38)	
Yes	6 (0.56)	4 (0.53)	2 (0.62)	
Metaraminol, *n* (%)				0.434
No	1,063 (99.35)	745 (99.47)	318 (99.07)	
Yes	7 (0.65)	4 (0.53)	3 (0.93)	
Reflux misabsorption, *n* (%)				1.000
No	1,069 (99.91)	748 (99.87)	321 (100.00)	
Yes	1 (0.09)	1 (0.13)	0 (0.00)	
Nausea, *n* (%)				0.247
No	1,019 (95.23)	717 (95.73)	302 (94.08)	
Yes	51 (4.77)	32 (4.27)	19 (5.92)	
Emesis, *n* (%)				0.173
No	1,051 (98.22)	733 (97.86)	318 (99.07)	
Yes	19 (1.78)	16 (2.14)	3 (0.93)	
Dysphoria, *n* (%)				0.761
No	1,036 (96.82)	726 (96.93)	310 (96.57)	
Yes	34 (3.18)	23 (3.07)	11 (3.43)	
Ventosity, *n* (%)				0.740
No	784 (73.27)	551 (73.57)	233 (72.59)	
Yes	286 (26.73)	198 (26.43)	88 (27.41)	
Hypoglycemia, *n* (%)				0.572
No	1,055 (98.60)	737 (98.40)	318 (99.07)	
Yes	15 (1.40)	12 (1.60)	3 (0.93)	
Giddy, *n* (%)				0.128
No	719 (67.20)	514 (68.63)	205 (63.86)	
Yes	351 (32.80)	235 (31.37)	116 (36.14)	
gastrointestinal endoscopy, *n* (%)				0.955
Gastroscopy only	412 (38.51)	289 (38.59)	123 (38.32)	
Colonoscopy only	114 (10.65)	81 (10.81)	33 (10.28)	
Gastroscopy and colonoscopy	544 (50.84)	379 (50.60)	165 (51.40)	
Adverse events, *n* (%)				0.839
No	967 (90.37)	676 (90.25)	291 (90.65)	
Yes	103 (9.63)	73 (9.75)	30 (9.35)	

### 3.3 Model variable selection

**Figure 2:**
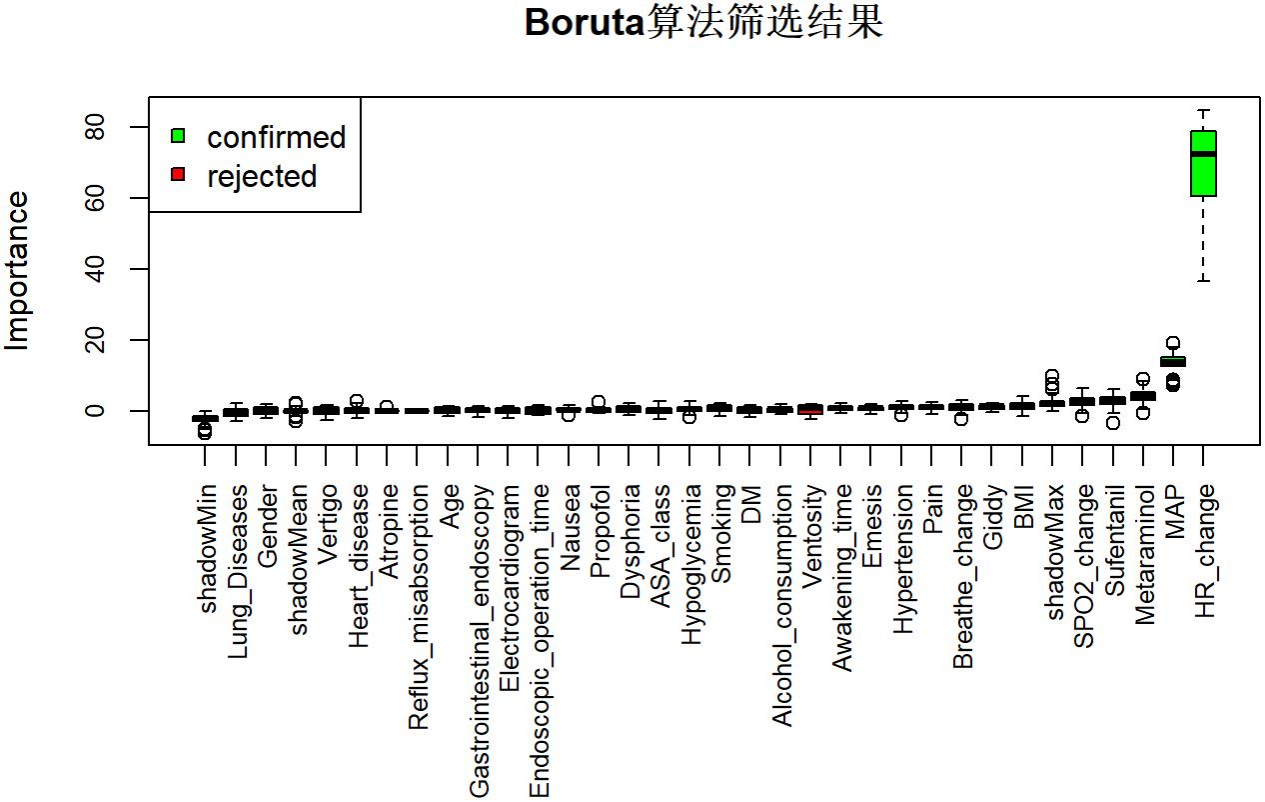
Image of the boruta method for selecting the ML model variables.

This study subsequently employed the Boruta algorithm with shadow features to identify five potentially effective predictor variables (corresponding to the green modules in Figure 2). Machine learning models were trained and constructed using these shadow feature variables, including HR change, MAP, metaraminol, sufentanil, and SpO_2_ change.

### 3.4 Model evaluation and comparison

**Figure 3:**
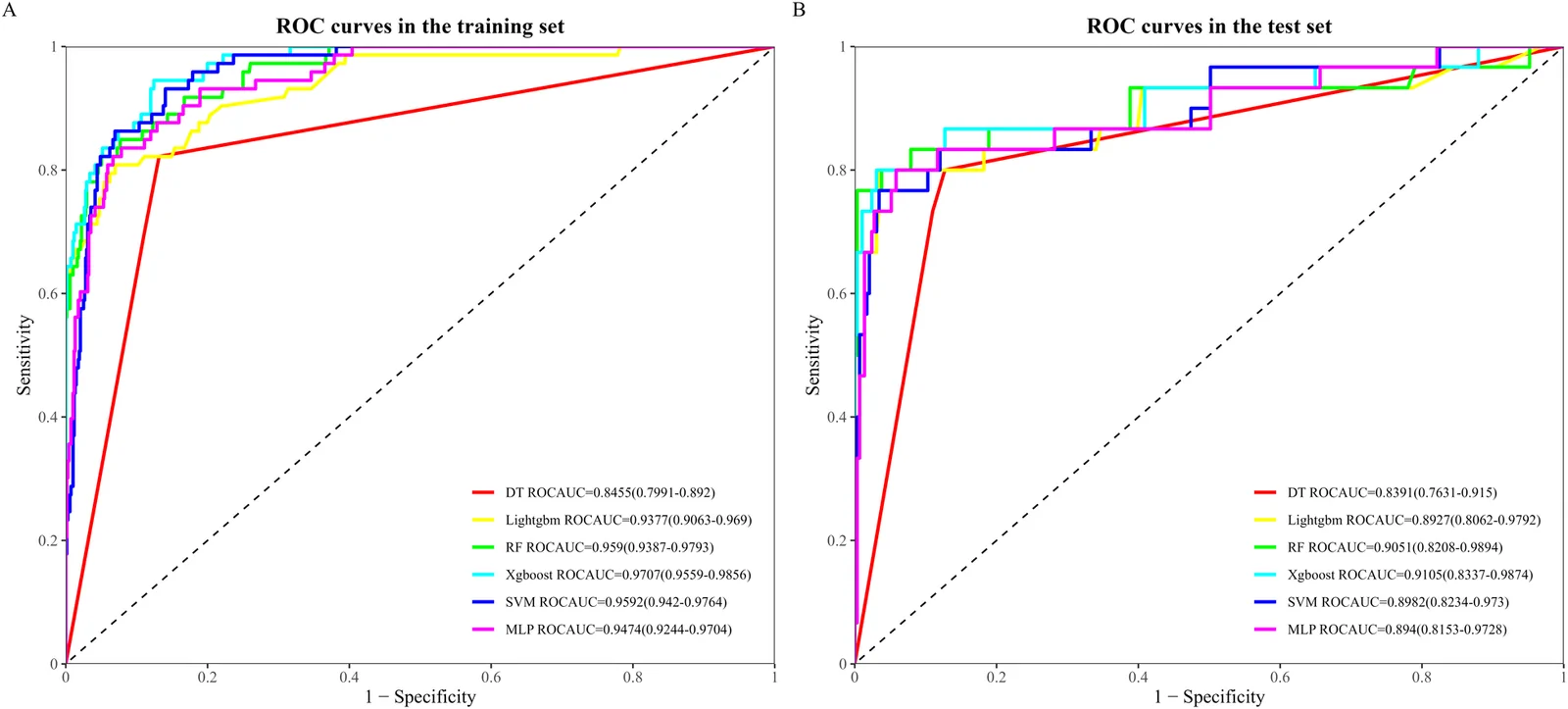
ROC-AUC of the test and validation sets of the 6 ML models. **(A)** ROC-AUC of the validation set (5-Fold Cross-Validation). **(B)** ROC-AUC of the validation sets.

**Figure 4:**
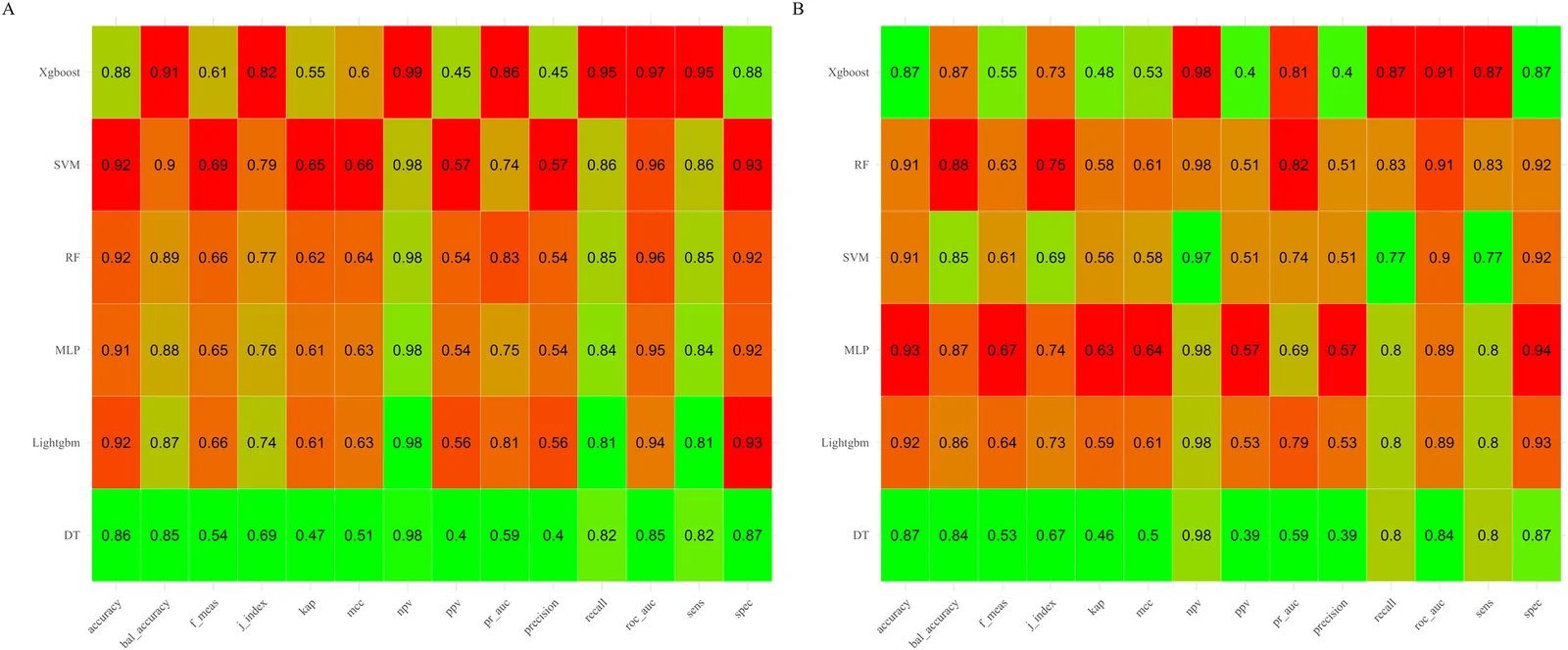
Performance comparison of different machine learning models on the training (5-fold cross-validation) **(A)** and validation **(B)** sets across multiple evaluation metrics. The heatmaps display the performance of the RF, Lightgbm, SVM, XGBoost, MLP, and DT models. Each cell represents the value of a specific evaluation metric, including accuracy, balanced accuracy, F1 score, J-index, kappa, Matthew's correlation coefficient (MCC), positive predictive value (PPV), negative predictive value (NPV), precision, recall, ROC AUC, sensitivity (sens), and specificity (spec). Higher values are indicated in red, whereas lower values are represented in green, indicating the model's effectiveness in both the training and validation sets.

Figure 3 presents the ROC-AUC of the six ML models —RF, LightGBM, DT, XGBoost, MLP, and SVM—in the training (Figure 3A) and independent validation sets (Figure 3B). XGBoost achieved the highest cross-validated ROC-AUC in the training set and was therefore selected as the final model before evaluation of the validation sets. Its ROC-AUC was 0.971 (95% CI, 0.956–0.986) in the training set and 0.910 (95% CI, 0.834–0.987) in the validation sets. Figure 4 summarizes the threshold-dependent performance of the candidate models in the training (Figure 4A) and validation sets (Figure 4B), with the corresponding numerical results presented in Supplementary Table S4.

**Figure 5:**
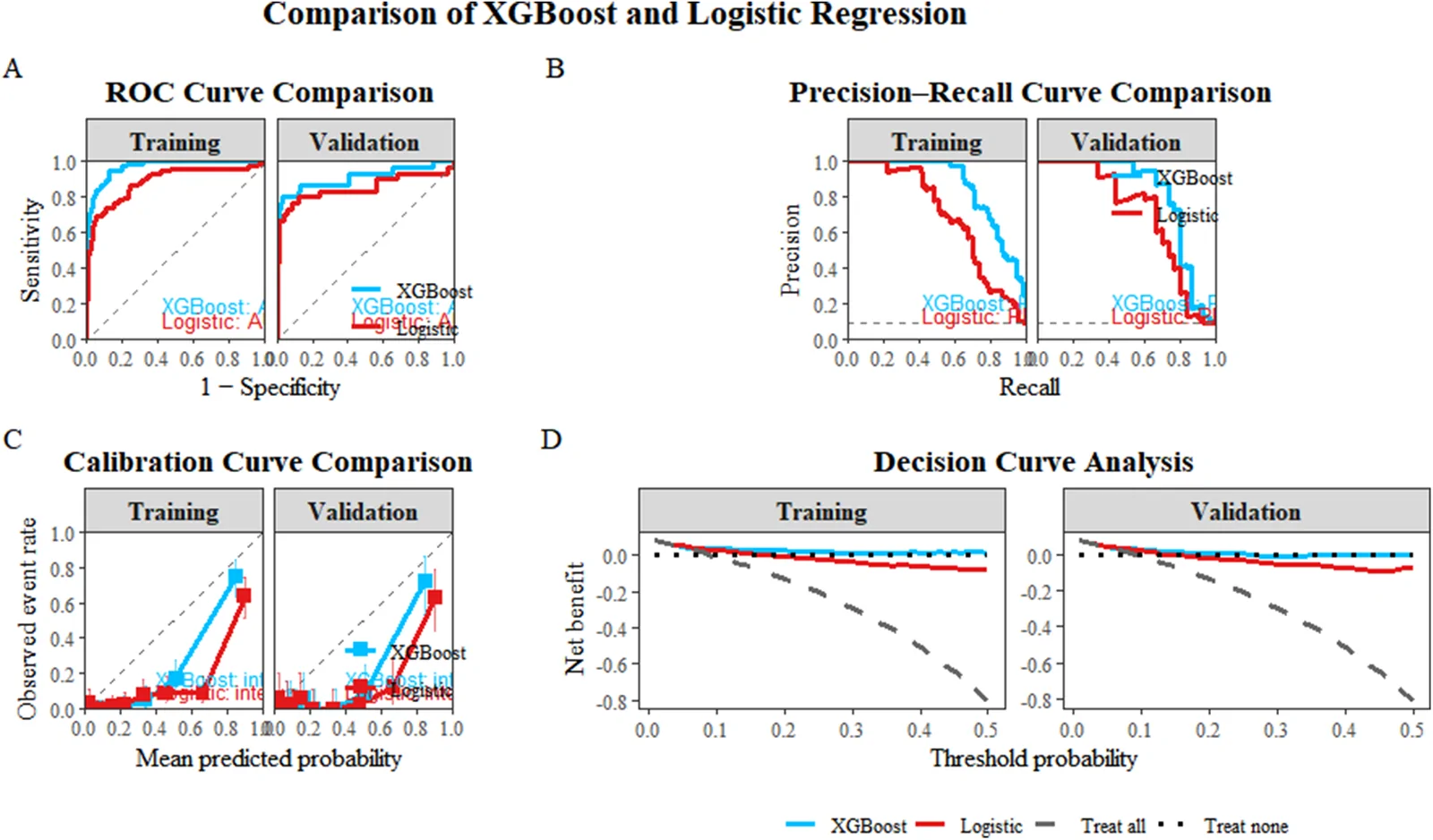
Comparison of the conventional logistic regression model and XGBoost in the training (5-fold cross-validation) and validation set. **(A)** ROC-AUC. **(B)** PR-AUC. **(C)** Calibration curves. **(D)** DCA.

A conventional multivariable logistic regression model incorporating the same five predictors as XGBoost was additionally developed as a reference model. In the validation set, the ROC-AUC was (0.852; 95% CI, 0.738–0.943) for logistic regression and 0.910 (95% CI, 0.834–0.987) for XGBoost. The corresponding PR-AUC values were 0.815 and 0.706, respectively. Complete discrimination and classification metrics are presented in Figure 5 and Supplementary Table S4.

Calibration was assessed using calibration plots, Brier scores, calibration intercepts, and calibration slopes. In the validation set, the Brier score, calibration intercept, and calibration slope were 0.076, −1.760, and 1.480 for XGBoost and 0.125, −2.250, and 0.947 for logistic regression, respectively. PR-AUC and DCA analyses further showed that XGBoost provided generally greater net benefit than logistic regression across threshold probabilities of 10%–50%.

The complete ROC-AUC, PR-AUC, calibration, and DCA for all candidate ML models are provided in Supplementary Figures S1–S3, together with the corresponding Brier scores, calibration intercepts, calibration slopes, and classification metrics in Supplementary Table S4. Overall, the incremental value of XGBoost over conventional logistic regression was evaluated jointly according to discrimination, calibration, and clinical utility. SHAP analysis was subsequently performed to characterize the contributions of the five predictors to the final XGBoost model.

### 3.5 Visualization of feature importance

**Figure 6:**
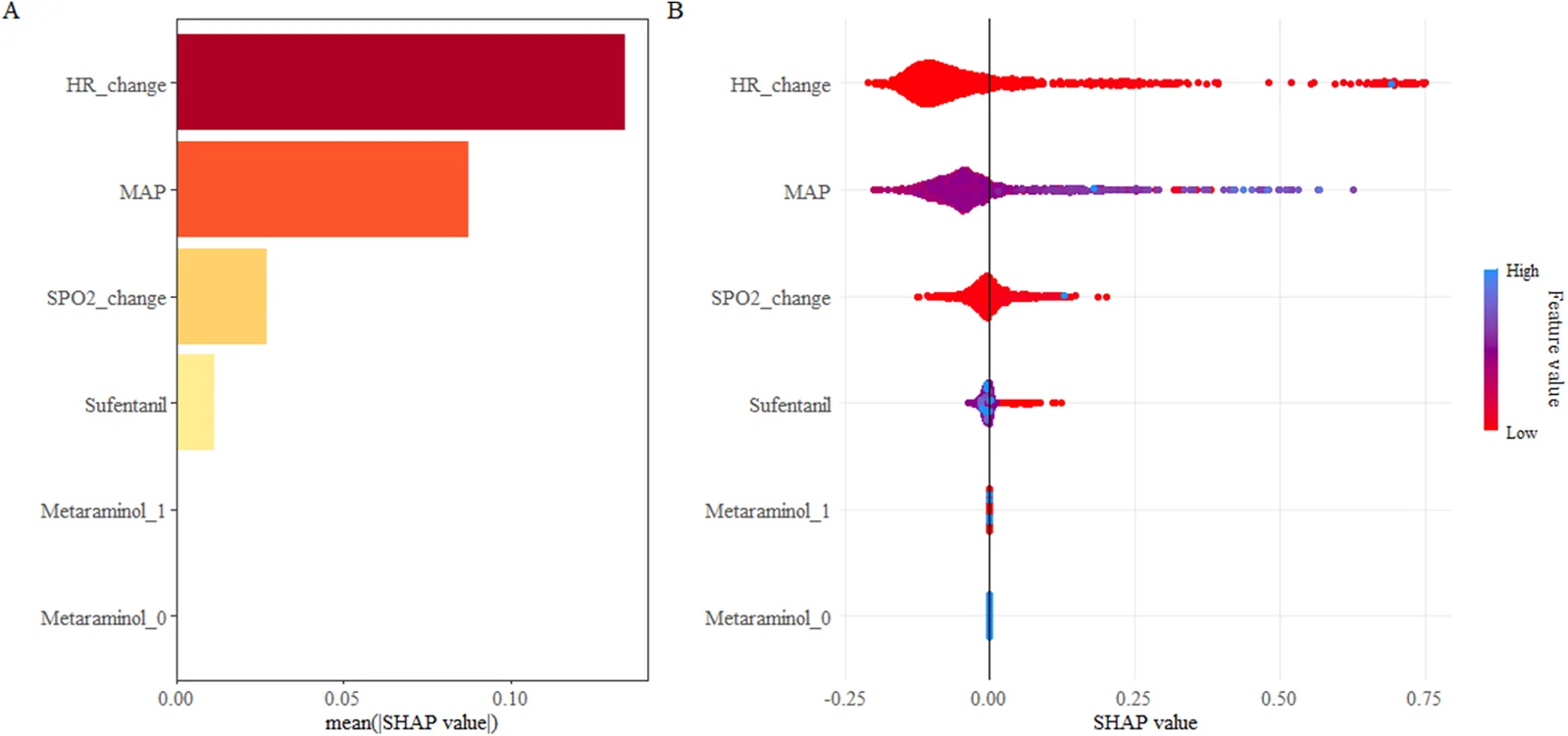
SHAP-based interpretation of the final XGBoost model. **(A)** Global feature importance ranked according to the mean absolute SHAP value. **(B)** SHAP summary plot showing the direction and magnitude of each predictor's contribution to the model output. Positive and negative SHAP values indicate contributions toward higher and lower model-predicted probabilities of subsequent AEs, respectively. Red indicates higher feature values, and blue indicates lower feature values. SHAP values represent model-based predictive associations and should not be interpreted as causal effects or treatment recommendations.

We performed SHAP analysis to assess the relative importance of each feature and its contribution to predictions generated by the final XGBoost model (Figures 6A,6B). In the SHAP summary plot, colors represent the observed feature values, with red indicating higher values and blue indicating lower values. HR change had the largest mean absolute SHAP value, followed by MAP, SpO_2_ change, sufentanil, and metaraminol. Positive SHAP values indicated contributions toward a higher model-predicted probability of subsequent AEs, whereas negative values indicated contributions toward a lower model-predicted probability. Overall, the model predictions were influenced primarily by HR change, MAP, SpO_2_ change, and sufentanil, while metaraminol had the smallest mean absolute SHAP value among the five predictors. These findings describe the internal predictive behavior of the fitted model and should not be interpreted as causal effects.

### 3.6 Sensitivity analyses

Among the 1,070 included patients, 103(9.6%) experienced at least one AE during the >15–30 min post-awakening outcome window. Respiratory and hemodynamic AEs occurred in 2 (1.94%) and 101 (98.06%) patients, respectively, with 0 patients experiencing both event types. Detailed subtype-specific distributions are provided in Supplementary Table S2.

Given the occurrence of only two respiratory events, these events were summarized descriptively and were not modeled separately. When the primary XGBoost model was re-evaluated using hemodynamic AEs alone as the outcome, the validation ROC-AUC was 0.930 (95% CI, 0.861–0.999), compared with 0.911 (95% CI, 0.834–0.987) for the composite outcome (*Δ*AUC = 0.019). These findings indicated broadly consistent discrimination and suggested that the primary model performance was largely driven by hemodynamic events (Supplementary Table S5).

## 4 Discussion

This study compared six ML algorithms for predicting subsequent AEs in patients undergoing sedated GI endoscopy in the PACU. XGBoost achieved the best cross-validated performance, with ROC-AUCs of 0.971 in the training set and 0.910 in the independent validation set. Compared with a conventional logistic regression model using the same five predictors, XGBoost demonstrated superior discrimination, better calibration, as reflected by the Brier score, calibration intercept, and calibration slope, and greater net clinical benefit on DCA. These findings support the incremental value of XGBoost beyond a conventional linear modelling approach, potentially owing to its ability to capture nonlinear relationships and interactions among predictors. SHAP analysis further clarified the contribution of individual predictors to the model-generated risk estimates, thereby improving model transparency. However, SHAP values represent model-based associations rather than causal effects. Accordingly, the model may support risk-adapted PACU monitoring after sedated GI endoscopy, but external validation and prospective clinical evaluation are required before routine implementation.

The clinical scope of the present model differs fundamentally from that of emerging ML approaches for gastric cancer detection. Plasma metabolomic, tongue-coating metaproteomic, and optimized classification models may facilitate non-invasive or minimally invasive screening and referral prioritization (1–3). However, these approaches address cancer detection rather than procedural safety and do not replace endoscopy when direct mucosal visualization, targeted biopsy, histopathological confirmation, or endoscopic treatment is required (5). Our model applies at a later stage of care, after patients have undergone sedated GI endoscopy, and estimates the risk of post-awakening AEs to support risk-adapted PACU monitoring rather than cancer screening or diagnostic decision-making.

XGBoost, as an efficient gradient boosting method, is well suited for nonlinear and high-dimensional sparse data. It suppresses overfitting through regularization terms and accelerates training via parallel computation, enabling outstanding performance in the data environment of this study. Although machine learning is often criticized as a “black box,” SHAP enables us to quantify the direction and strength of each feature's contribution to both individual predictions and the overall model (33). This not only enhances interpretability but also provides clinicians with intuitive physiological insights, thereby bridging the trust gap between algorithms and clinical practice. Importantly, SHAP provides associative explanations rather than causal proof. Clinical application should incorporate medical history and physiological judgment.

Feature selection and model interpretation are important components of clinical prediction modelling. SHAP analysis identified HR change, MAP, SpO_2_ change, sufentanil, and metaraminol as the main variables contributing to predictions generated by the final XGBoost model. However, SHAP values describe model-based associations and feature contributions rather than causal relationships. Therefore, these findings should be interpreted as explanations of model behavior rather than evidence supporting clinical interventions.

First, HR changes made the most significant contribution in this study. HR is a readily available physiological parameter that may reflect changes in autonomic nervous system activity, sedation status, and hemodynamic conditions, including intravascular volume status. Abnormal HR fluctuations may signal pathological processes such as variations in sedation depth, enhanced vagal reflexes, volume depletion, or arrhythmias. In many cases, an abnormal HR precedes a significant decrease in blood pressure or oxygenation deterioration (34). Therefore, continuous HR monitoring and trend analysis aid in the early identification of patients at risk for circulatory instability, triggering timely assessment and intervention. In practical applications, combining short-term HR mutations with long-term trend indicators into early warning thresholds can reduce missed diagnosis rates.

Second, the significance of the MAP is directly linked to tissue perfusion and organ function (35). Previous evidence suggests that transient or recurrent MAP decreases—particularly below 60–70 mmHg—may increase the risk of myocardial and renal injury (36). The high SHAP values for MAP in this study underscore that even minor blood pressure fluctuations may impact postoperative outcomes in short-acting analgesia endoscopy settings. Clinically, this implies that relying solely on absolute low-pressure thresholds may be insufficient to capture harmful hypoperfusion events in individual patients. Instead, a more nuanced intervention strategy should consider both relative baseline decreases and the patient's underlying cardiovascular vulnerability.

Furthermore, changes in SpO_2_ serve as a direct indicator of respiratory function. Its significance within the model reflects the central role of respiratory depression or hypoventilation in the occurrence of sedation-related AEs (37). Sedated endoscopy commonly employs combined sedation with propofol and opioids, which synergistically suppress the respiratory center, leading to hypoventilation, carbon dioxide retention, and hypoxic events. These alterations can further amplify circulatory instability by affecting HR and blood pressure. Therefore, continuous pulse oximetry and respiratory monitoring, supplemented by capnography when clinically indicated, may facilitate the early recognition of respiratory deterioration and inform the timely escalation of oxygen therapy or ventilatory support.

With respect to medication-related predictors, sufentanil and metaraminol showed different contributions to the model predictions. Sufentanil, a potent µ-opioid receptor agonist, provides effective analgesia but may also be associated with respiratory depression and hemodynamic suppression (38, 39). However, its predictive contribution may reflect patient characteristics, procedural conditions, and clinician dosing decisions rather than a causal dose-response relationship. Metaraminol increases peripheral vascular resistance and is commonly administered in response to hypotension or vasodilation (40). Therefore, its association with a lower model-predicted probability of subsequent AEs may reflect preceding hemodynamic instability, clinician recognition and treatment, treatment response, confounding by indication, or other unmeasured factors rather than a direct protective effect. Accordingly, these findings should not be used to support specific sufentanil dosing or metaraminol treatment strategies.

On the basis of these findings, we recommend enhancing continuous and trend-based monitoring of HR, MAP, and SpO_2_ in the PACU. Combining real-time machine learning risk scores with these critical physiological parameters can serve as an auxiliary guide for triggering further assessment or intervention. For example, when abnormal HR fluctuations occur alongside a relative decrease in the MAP from baseline, sedation depth should be adjusted, low-dose vasopressors should be initiated, or goal-directed fluid therapy should be implemented. If SpO_2_ shows a persistent decline, airway and oxygenation strategies should be prioritized, and respiratory support should be promptly initiated. Following external validation, interpretable models may be prospectively evaluated for integration into PACU monitoring systems to support early warning, risk-adapted monitoring, and timely clinical assessment. The present study did not evaluate model-triggered interventions, feedback mechanisms, or improvements in clinical outcomes.

## 5 Limitations

This study has several limitations. First, the temporal structure limits interpretation of the model as strictly prospective. HR and SpO_2_ changes were derived from measurements obtained during 15–30 min after awakening, overlapping with the outcome assessment window; therefore, they may reflect evolving physiological instability rather than predictors available before outcome occurrence. The findings should thus be interpreted as early risk identification. Moreover, AEs occurring within the first 15 min after awakening were not included, limiting the model's applicability to the immediate post-awakening period. Future studies should use temporally separated predictor and outcome windows or dynamic prediction approaches. Second, this was a single-center study with internal validation from the same clinical setting, which may limit model transportability. Temporal and independent multicenter external validation are therefore required. Third, the observational design precludes causal inference. SHAP values indicate predictor contributions to model-estimated risk rather than causal effects, and medication-related variables may reflect clinical responses to preceding physiological instability. Prospective impact studies are needed to assess clinical utility. Fourth, KNN imputation and Boruta feature selection were performed on the complete training dataset before cross-validation, potentially introducing optimism into model selection, although the validation set remained isolated. The limited sample size and number of events may also have affected model stability, while SMOTENC-generated observations may not fully represent real-world distributions. Future studies should incorporate all data-dependent preprocessing within resampling and evaluate the model in larger independent cohorts.

## 6 Conclusion

In summary, among patients who remained free of qualifying AEs at 15 min after awakening from sedated GI endoscopy, the XGBoost model demonstrated promising predictive performance in predicting new-onset PACU AEs occurring between 15 and 30 min in this single-center cohort and validation set. SHAP analysis identified HR change, MAP, SpO_2_ change, sufentanil dose, and metaraminol use as variables contributing to the model predictions; however, these associations should not be interpreted as causal effects or treatment recommendations. These preliminary findings support further evaluation of the model as a potential tool for early risk stratification. Temporal and independent multicenter external validation, together with assessment of calibration, transportability, and clinical impact, is required before the model can be considered for integration into routine clinical workflows.

## Data Availability

The raw data supporting the conclusions of this article will be made available by the authors, without undue reservation.
