# Supplementary material for: Machine learning prediction of adverse events during minutes 15–30 of recovery in patients event-free at 15 min after sedated gastrointestinal endoscopy
Source: Front Med (Lausanne). 2026 Aug 27;13:1930976. doi: 10.3389/fmed.2026.1930976 (PMC13558014; doi:10.3389/fmed.2026.1930976)
Supplement: Supplementary file 1 [file Datasheet1.pdf]

Supplementary material

Supplementary Table S1 Definitions and calculation formulas for changes in physiological parameters

variable	calculation formula
HR change	HR $change_{30min}(\%)=\frac{HR_{30\min}-HR_{15\min}}{HR_{15\min}}\times 100\%$
SpO_2 change	SpO_2 $change_{30min}(\%)=\frac{SpO_{230\min}-SpO_{215\min}}{SpO_{215\min}}\times 100\%$
Breathe change	Breathe change30min(%)= $\frac{\text{Breathe change}_{30min}-\text{Breathe change}_{15min}}{\text{Breathe change}_{15min}}\times 100\%$

Supplementary Table S2 Distribution of adverse events during the prespecified outcome window

Outcome	Total（n=103）	Training(n =73)	validation(n = 30)
Composite adverse event, n(%)	103,(100.00%)	73,(100.00%)	30,(100.00%)
Respiratory depression, n(%)	2,(1.94%)	1,(1.37%)	1(3.33%)
hemodynamic event, n(%)	101,(98.06%)	72,(98.63%)	29,(96.67%)
Concurrent respiratory depression and hemodynamic AEs, n(%)	0,(0.00%)	0,(0.00%)	0,(0.00%)

Supplementary Table S3 Hyperparameter search spaces and selected values for the candidate machine-learning models

Model	Engine	Tuned hyperparameters and search ranges	Search strategy	Candidate combinations
DT	rpart	tree_depth: 3–7; min_n: 15–25; cost_complexity: 0.01–0.10(log10 范围−2 至 −1)	Random grid search	20
RF	ranger	mtry: 2–10; trees: 200–500; min_n: 155–205	Random grid search	20
XGBoost	xgboost	mtry: 2–8; min_n: 5–55; tree_depth: 3–8; learn_rate: 0.001–0.10(log10 范围−3 至−1); loss_reduction: 0.001–1(log10 范围−3 至 0); sample_size: 0.70–0.90	Random grid search	50
LightGBM	lightgbm	tree_depth: 1–3; trees: 100–500; learn_rate: 0.001–0.10; mtry: 2–8; min_n: 35–55; loss_reduction: 0.001–1	Random grid search	50
MLP	nnet	hidden_units: 15–24; penalty: 0.001–1(log10 范围−3 至 0); epochs: 50–150	Regular grid search, 3 levels per parameter	27
SVM	kernlab	cost: 0.03125–32(log2 范围−5 至 5); rbf_sigma: 0.0001–1(log10 范围−4 至 0)	Random grid search	30

Supplementary Table S4 Performance comparison of different machine learning models on the training and validation sets across multiple evaluation metrics

evaluation metrics	XGBoost		RF		LightGBM		SVM		MLP		DT		Logistic	
	**Training**	**validating**	**Training**	**validating**	**Training**	**validating**	**Training**	**validating**	**Training**	**validating**	**Training**	**validating**	**Training**	**validating**
AUC	0.971 (0.955-0.984)	0.911 (0.826-0.975)	0.959 (0.938-0.978)	0.905 (0.813-0.976)	0.938 (0.905-0.967)	0.893 (0.800-0.967)	0.959 (0.942-0.975)	0.898 (0.819-0.963)	0.947 (0.924-0.969)	0.894 (0.806-0.961)	0.846 (0.800-0.890)	0.839 (0.762-0.908)	0.889(0.836-0.936)	0.852 (0.738-0.943)
Accuracy	0.883 (0.858-0.905)	0.866 (0.829-0.900)	0.916 (0.896-0.935)	0.910 (0.879-0.938)	0.919 (0.899-0.937)	0.913 (0.882-0.944)	0.924 (0.905-0.941)	0.910 (0.875-0.938)	0.913 (0.893-0.933)	0.925 (0.897-0.953)	0.864 (0.840-0.888)	0.866 (0.829-0.900)	0.921(0.903-0.940)	0.919 (0.891-0.947)
sensitivity	0.945 (0.890-0.986)	0.867 (0.733-0.967)	0.849 (0.767-0.932)	0.833 (0.700-0.967)	0.808 (0.726-0.890)	0.800 (0.666-0.933)	0.863 (0.781-0.932)	0.767 (0.600-0.900)	0.836 (0.753-0.918)	0.800 (0.666-0.933)	0.822 (0.726-0.904)	0.800 (0.666-0.933)	0.699(0.603-0.808)	0.700 (0.533-0.867)
specificity	0.876 (0.851-0.901)	0.866 (0.828-0.904)	0.923 (0.902-0.942)	0.918 (0.887-0.945)	0.930 (0.910-0.950)	0.924 (0.893-0.955)	0.930 (0.911-0.948)	0.924 (0.893-0.955)	0.922 (0.901-0.941)	0.938 (0.911-0.966)	0.868 (0.842-0.893)	0.873 (0.835-0.911)	0.945(0.928-0.962)	0.942 (0.914-0.966)
PPV	0.451 (0.402-0.507)	0.400 (0.333-0.483)	0.544 (0.480-0.618)	0.510 (0.421-0.619)	0.557 (0.488-0.638)	0.522 (0.426-0.649)	0.573 (0.508-0.647)	0.511(0.400-0.634)	0.535 (0.472-0.608)	0.571 (0.468-0.703)	0.403 (0.353-0.460)	0.393 (0.319-0.482)	0.580(0.500-0.671)	0.553 (0.435-0.692)
NPV	0.993 (0.987-0.998)	0.984 (0.969-0.996)	0.983 (0.973-0.992)	0.982 (0.967-0.996)	0.978 (0.969-0.988)	0.978 (0.963-0.993)	0.984 (0.975-0.992)	0.975 (0.957-0.989)	0.981 (0.972-0.990)	0.978 (0.964-0.993)	0.978 (0.968-0.988)	0.977 (0.960-0.992)	0.967(0.956-0.978)	0.968 (0.951-0.986)
F1 score	0.611 (0.561-0.664)	0.547 (0.466-0.630)	0.663 (0.600-0.729)	0.633 (0.535-0.732)	0.659 (0.593-0.730)	0.632 (0.528-0.738)	0.689 (0.628-0.750)	0.613 (0.494-0.725)	0.652 (0.588-0.719)	0.667 (0.563-0.771)	0.541 (0.484-0.601)	0.527 (0.438-0.621)	0.634(0.558-0.716)	0.618 (0.492-0.738)
Brier Score	0.068 (0.059-0.076)	0.076 (0.062-0.092)	0.083 (0.072-0.093)	0.087 (0.079-0.105)	0.143 (0.137-0.149)	0.143 (0.134-0.152)	0.077 (0.063-0.091)	0.087 (0.066-0.112)	0.140 (0.133-0.146)	0.141 (0.132-0.152)	0.121 (0.103-0.138)	0.119 (0.094-0.146)	0.120(0.107-0.134)	0.125 (0.103-0.147)
Calibration intercept	-1.690(-1.810--1.570)	-1.760(-1.950--1.560)	-1.940(-2.120--1.780)	-2.010(-2.280--1.720)	-1.890(-1.920--1.860)	-1.940(-1.990--1.880)	-2.130(-2.400--1.850)	-2.310(-2.770--1.760)	-1.880(-1.920--1.840)	-1.920(-1.980--1.850)	-2.210(-2.360--2.040)	-2.230(-2.440--1.970)	-2.150(-2.310--1.990)	-2.250(-2.490--2.000)
Calibration slope	1.930 (1.680-2.340)	1.480 (1.130-2.220)	1.640 (1.350-2.220)	1.090 (0.744-1.980)	3.730 (3.220-4.560)	3.350 (2.570-5.020)	0.911 (0.761-1.160)	0.631 (0.446-1.999)	3.650 (3.080-4.590)	3.060 (2.270-4.700)	0.952 (0.801-1.170)	0.925 (0.695-1.280)	1.070(0.863-1.370)	0.947 (0.614-1.510)

Supplementary Table S5 Sensitivity analysis of the primary XGBoost model (Performance in the independent validation set)

Analysis	Outcome	Model	ROC-AUC (95% CI)	ΔAUC vs primary
Primary analysis	Composite adverse events	Primary XGBoost	0.911 (0.834–0.987)	Reference
Sensitivity analysis	Hemodynamic adverse events	Primary XGBoost	0.930 (0.861–0.999)	+0.019

Δ AUC was calculated as the ROC-AUC for the hemodynamic-only outcome minus that for the primary composite outcome.

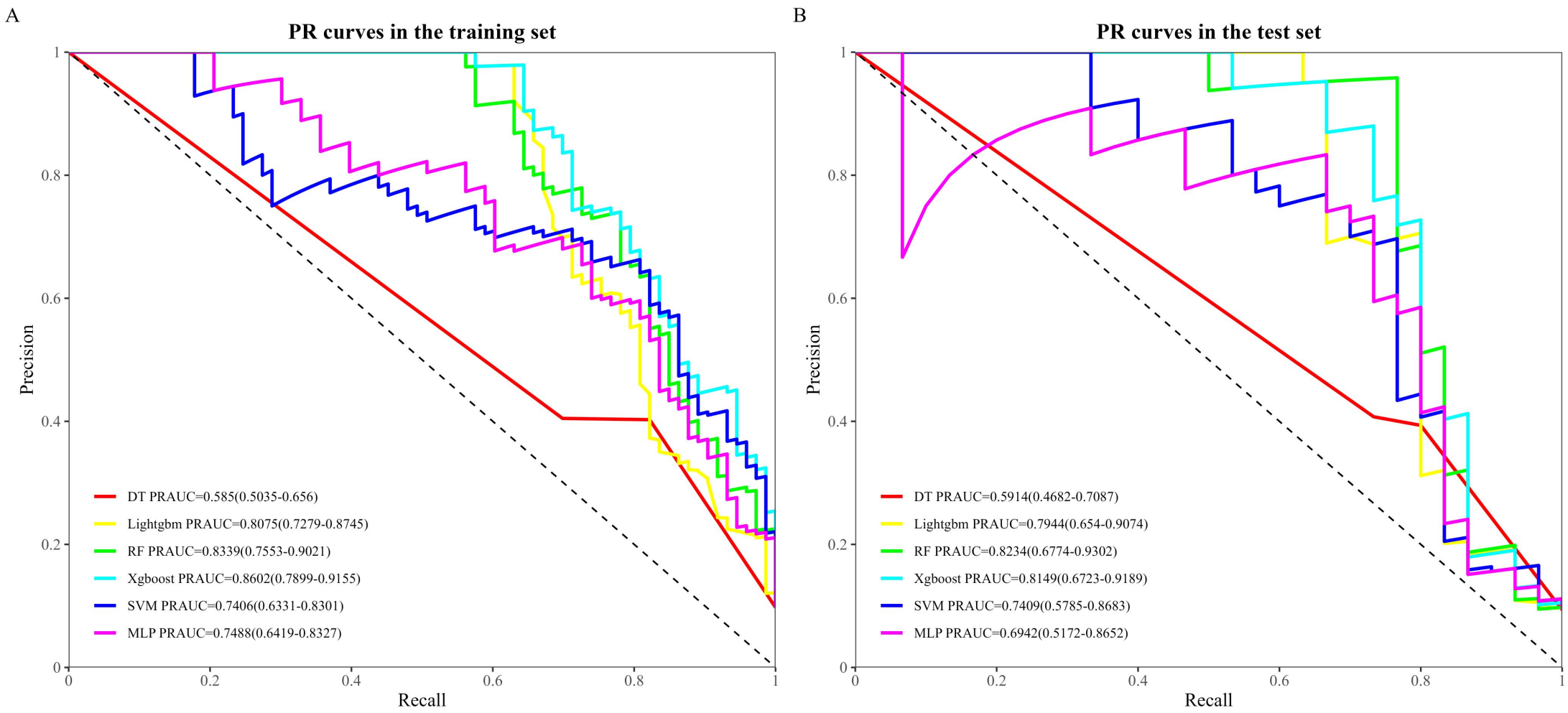

Supplementary Fig. S1 PR-AUC of the test and validation sets of the 6 ML models. (A) PR-AUC of the training set(5-Fold Cross-Validation). (B) PR-AUC of the validation sets.

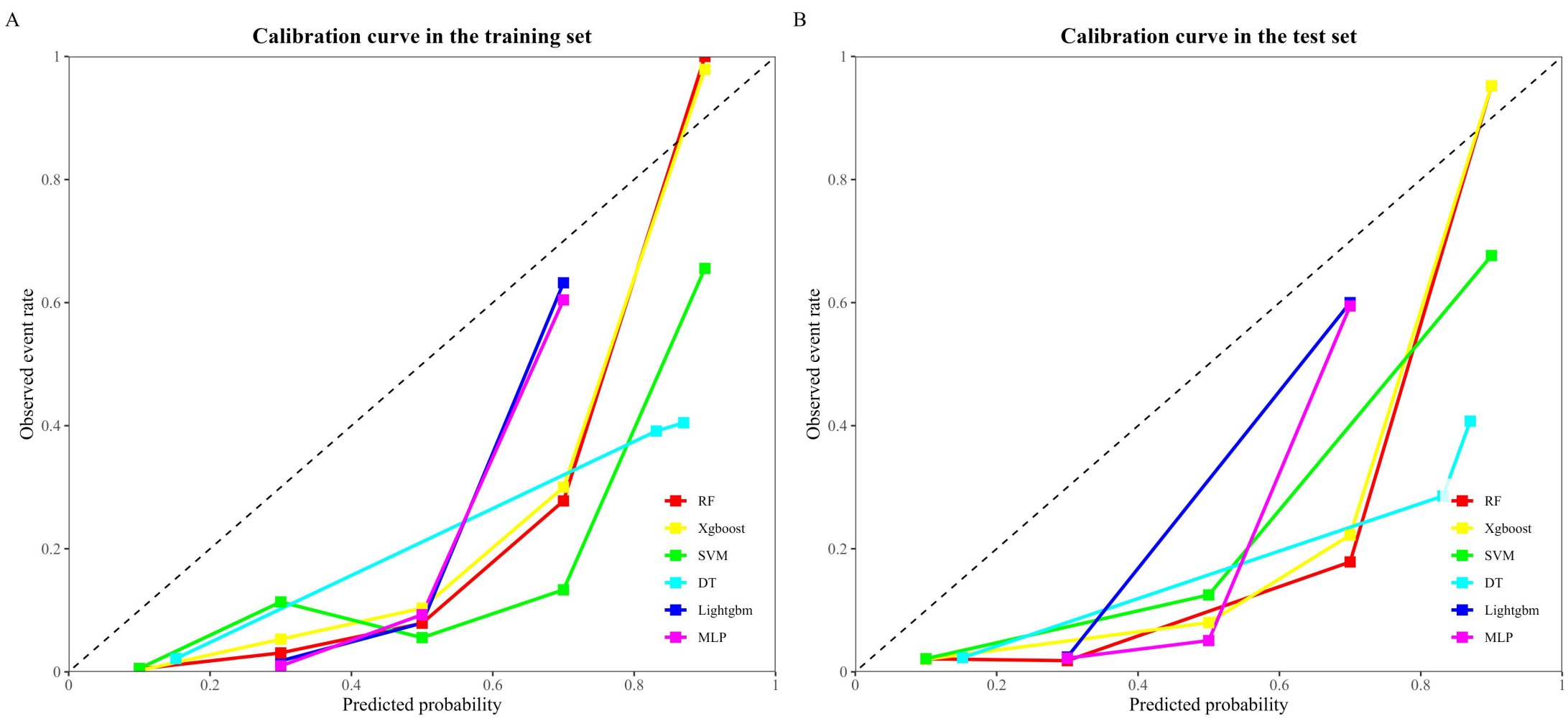

Supplementary Fig. S2 Calibration curves of the test and validation sets of the 6 ML models. (A) Calibration curves of the training set(5-Fold Cross-Validation). (B) Calibration curves of the validation sets.

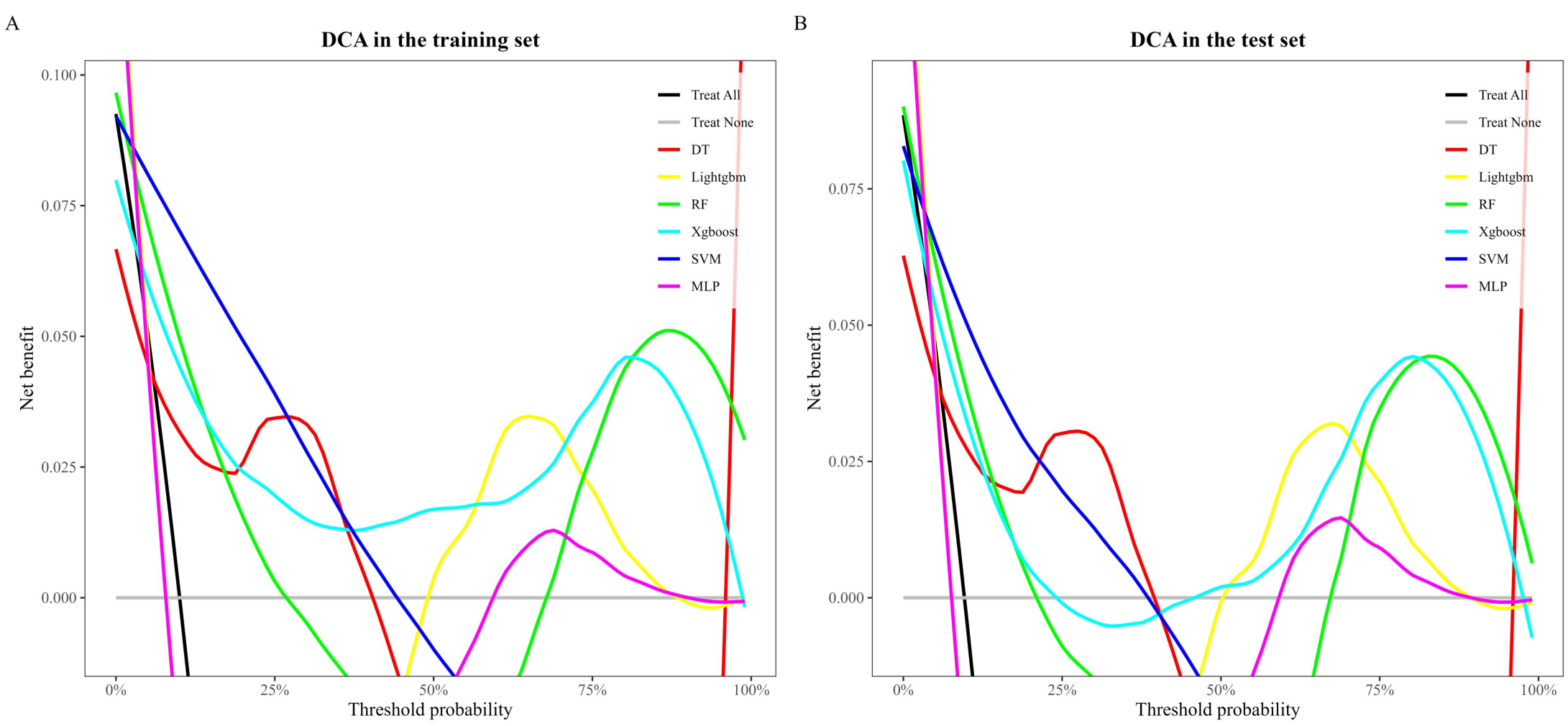

Supplementary Fig. S3 DCA of the test and validation sets of the 6 ML models. (A) DCA of the training set(5-Fold Cross-Validation). (B) DCA of the validation sets.
